# Rapidly Progressive Bilateral Vitreoretinal Lymphoma

**DOI:** 10.7759/cureus.31639

**Published:** 2022-11-18

**Authors:** Joseph W Fong, Riley N Sanders, David L Baker, Sergio Pina-Oviedo, Sami Uwaydat

**Affiliations:** 1 Department of Ophthalmology, University of Tennessee Health Science Center - Hamilton Eye Institute, Memphis, USA; 2 Department of Ophthalmology, University of Arkansas for Medical Sciences, Little Rock, USA; 3 Department of Ophthalmology, Baker Eye Institute, Conway, USA; 4 Department of Pathology, Duke University Hospital, Durham, USA; 5 Department of Pathology, University of Arkansas for Medical Sciences, Little Rock, USA

**Keywords:** vitreoretinal surgery, rapid progression lymphoma, ocular lymphoma, central nervous system lymphoma, primary vitreoretinal lymphoma

## Abstract

A 56-year-old male who presented with unilateral localized sub-retinal lesions suspicious for primary vitreoretinal lymphoma (PVRL) developed florid bilateral ocular involvement and was found to have lesions on MRI of the brain in a five-week period despite the absence of vitreous involvement during the entire course of his disease. His ocular lesions were monitored while on systemic treatment and an excellent clinical response was achieved. His central nervous system (CNS) lesions, however, continued to progress despite chemotherapy and whole-brain radiation. He died 12 months from his time of ocular diagnosis. To our knowledge, this case represents the most rapid progression of PVRL reported in the literature - from unilateral, localized lesions in the sub-retinal space to bilateral ocular involvement and identification of CNS involvement in a five-week period. This case highlights the potential for rapid ocular progression of PVRL stressing the need for early diagnosis. Therefore, we recommend prompt vitreous and, if necessary, sub-retinal biopsy in cases of suspected vitreoretinal lymphoma in addition to neuro-imaging. We emphasize the importance of coordination between pathologists, ophthalmologists, and oncologists for prompt, accurate diagnosis. Delay in diagnosis and treatment can result in rapid intraocular progression and central nervous system spread.

## Introduction

Primary vitreoretinal lymphoma (PVRL) is often considered a subset of primary central nervous system (CNS) lymphoma that follows a particularly aggressive clinical course and can be challenging to diagnose due to its ability to masquerade as other more common causes of uveitis [1]. The differential diagnosis includes infectious etiologies such as viral retinitis, Bartonella, tick-borne infections, tuberculosis, and syphilis, and non-infectious etiologies such as sarcoidosis, acute posterior multifocal placoid pigment epitheliopathy (APMPPE), and Behcet’s disease. High index of clinical suspicion for vitreoretinal lymphoma, with careful consideration of the patient's immune status and the characteristics of intraocular inflammation, requires prompt biopsy for quick and accurate diagnosis to prevent rapid intraocular progression and further CNS involvement [1,2]. We herein report a case of large B-cell vitreoretinal lymphoma that initially presented with unilateral involvement and within one month had undergone rapid progression in the affected eye. In addition, he developed new involvement of the previously unaffected eye and brain lesions were identified on neuro-imaging.

## Case presentation

**Figure 1:**
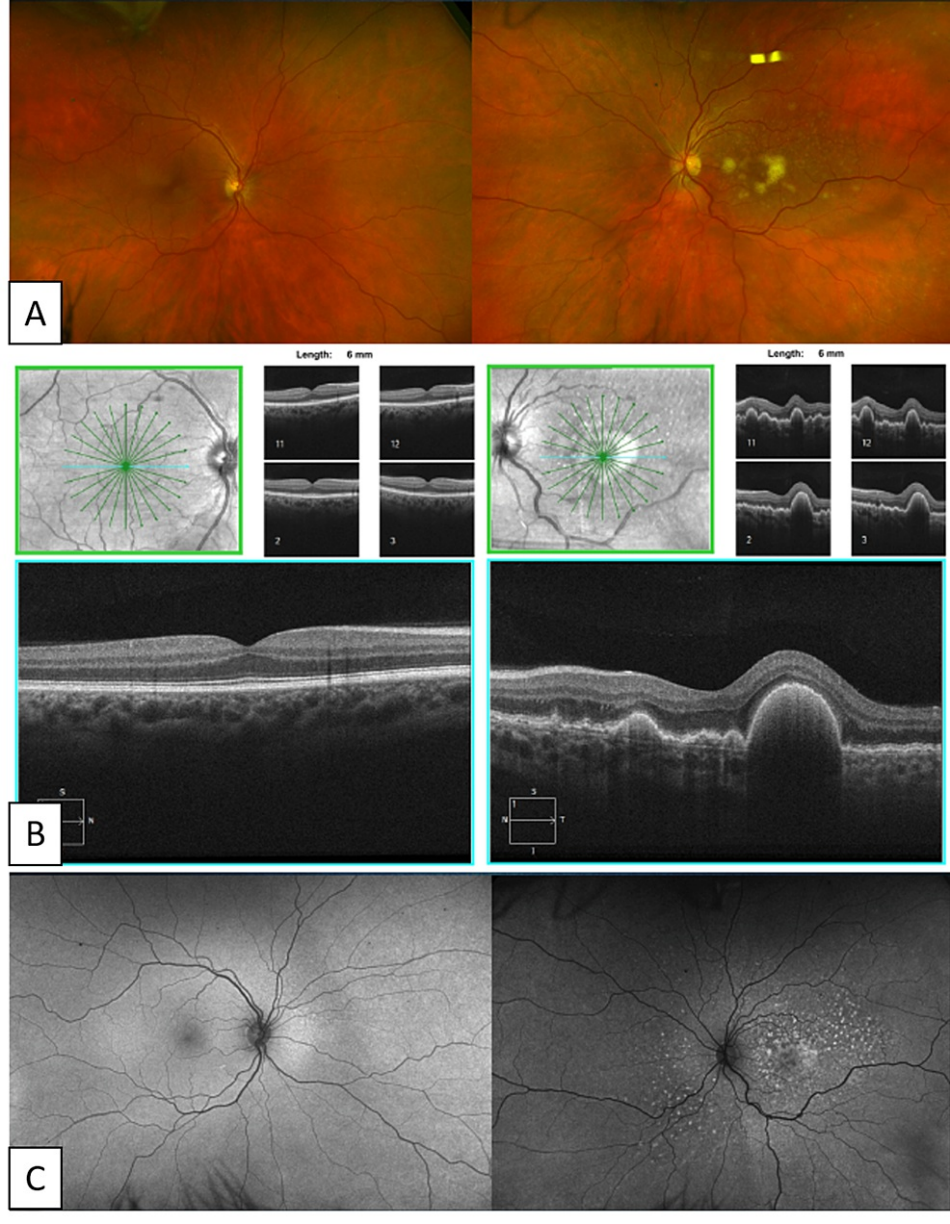
Initial Presentation On initial presentation, the left fundus reveals multiple creamy yellow retinal lesions (A) that were localized to the sub-RPE space by OCT (B). Autofluorescence (C) and OCT demonstrate a lack of involvement of the right eye. OCT: Optical coherence tomography

A 56-year-old white male sought medical attention for a two-month history of blurred vision and decreased visual acuity in the left eye. He had cataract surgery in the same eye three months prior with a normal fundus exam at all pre-operative visits and reported a good vision in the month following surgery. He denied any other neurologic symptoms and his past medical history was non-revealing. At the time of initial presentation, his uncorrected distance visual acuity (VA) was 20/20 in the right eye and 20/50 in the left eye. No proptosis was present. The anterior segment exam was within normal limits in both eyes. On posterior segment exam, he was found to have a normal fundus in the right eye and creamy yellow lesions in the left eye that were localized to the sub-retinal pigmented epithelial space (sub-RPE) by optical coherence tomography (Zeiss Cirrus 5000, Dublin, CA, USA) (Figure 1A and Figure 1B). The vitreous was noted to be clear and completely acellular. Fluorescein angiography and autofluorescence of the right eye (Optos California system, Optos, Dunfermline, Scotland) were normal. Fluorescein angiography of the left eye showed multiple areas of staining in macula and periphery, and autofluorescence showed patchy hypo- and hyper-autofluorescence (Figure 1C). Cervical, preauricular, supraclavicular, and axillary lymph nodes were palpated and revealed no adenopathy. Workup for infectious etiologies, including syphilis, tuberculosis, human immunodeficiency virus, West Nile, Coxsackie A9, Bartonella, Toxoplasma, and tick serologies, and workup for inflammatory etiologies, including angiotensin-converting enzyme and lysozyme, were ordered and were unremarkable. Cerebrospinal fluid (CSF) studies and magnetic resonance imaging (MRI) of the orbits ordered by a medical oncologist were normal and a full neurologic examination was unrevealing. An MRI of the brain was not obtained at this time by the medical oncologist, but limited cuts of the brain that were available did not demonstrate the presence of any lesions. Of note, the CSF cytology did not reveal the presence of malignant cells.

**Figure 2:**
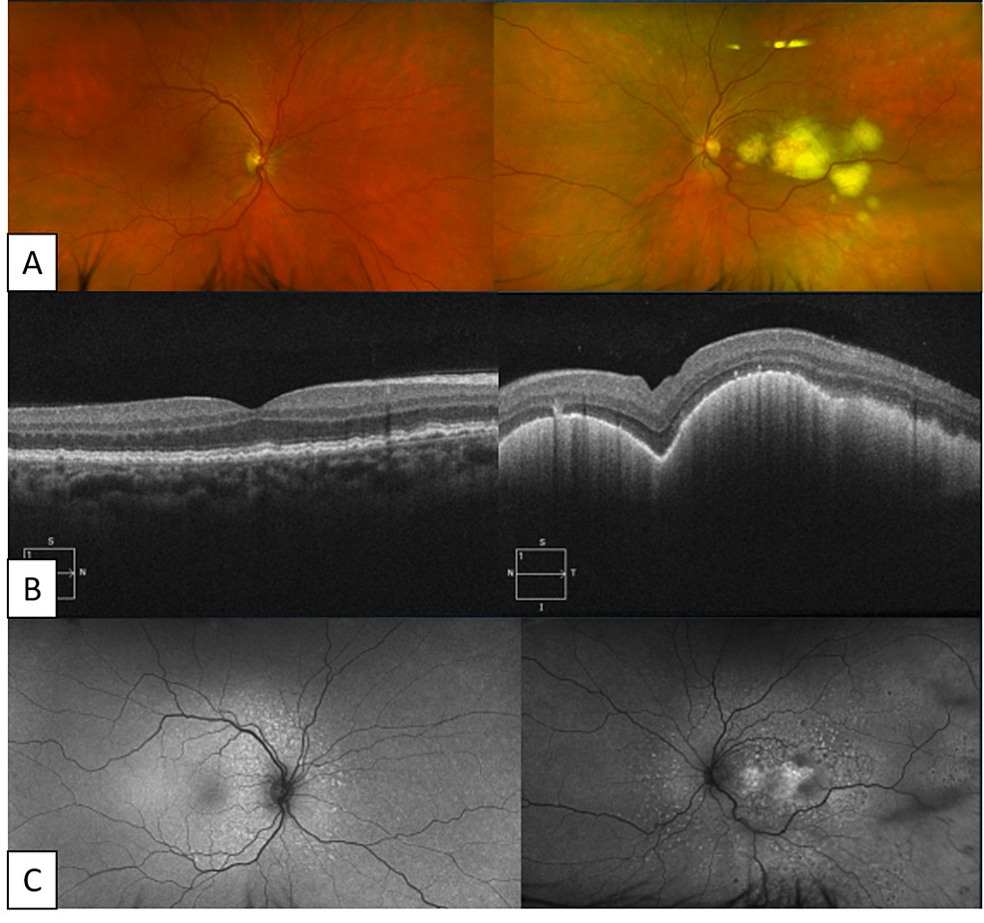
Five Weeks Later (A) Repeat exam of the right fundus reveals no visible retinal lesions one month after the initial presentation, but the left fundus shows a rapid progression of lesions. Repeat OCT (B) and autofluorescence (C) demonstrated new sub-RPE lesions in the previously unaffected right eye and the progression of lesions in the left eye. OCT: Optical coherence tomography

**Figure 3:**
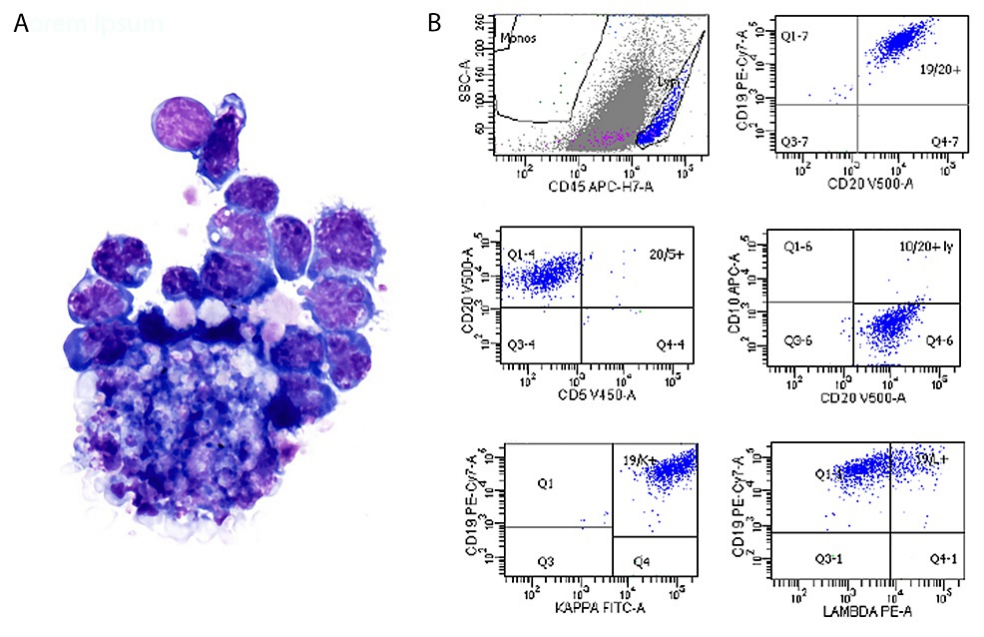
Cytospin Preparation and Flow Cytometry Immunophenotyping of Sub-Retinal Biopsy Specimen (A) Cytospin preparation of the sub-retinal lesion. There are large atypical lymphoid cells with oval to convoluted nucleus and moderate amount of basophilic cytoplasm. These cells are associated with cell debris and necrosis, as seen in the lower aspect of the image (Wright-Giemsa stain, 60X). (B) Flow cytometry immunophenotyping of the sub-retinal lesion. Top: The large lymphoma cells have intermediate to high scatter and are bright for CD45 (blue population). The large population in gray consisted of cellular debris and had non-specific staining with the other antibodies (not shown). The lymphoma cells are positive for CD19 and CD20, confirming their B-cell lineage. Middle panel: These cells are negative for CD5 and for CD10. Bottom panel: The lymphoma cells demonstrate kappa light chain restriction.

The patient missed his one-week follow-up appointment but returned to our clinic five weeks later and was found to have involvement of his previously unaffected right eye and significant progression of the creamy yellow retinal lesions of the left eye (Figure 2A-2C). The vitreous remained clear and acellular. A 23-gauge pars plana vitrectomy (PPV) with aspiration of the sub-retinal deposits was performed. To access the sub-retinal space, a small retinotomy was made over a large sub-retinal lesion. A 23/38-gauge PolyTip® catheter was then inserted into the sub-retinal space and careful aspiration was used to acquire a sizable biopsy specimen. The specimen was then immediately transported to the flow cytometry lab in a balanced salt solution. Microscopic evaluation of the sub-retinal aspirate showed medium-to-large atypical lymphoid cells with oval to convoluted nucleus and abundant necrotic debris (cytospin slide; Wright-Giemsa stain), and the concurrent flow cytometry analysis was positive for a CD5-negative/CD10-negative kappa-monotypic B-cell population with intermediate to high side scatter, consistent with large B-cell lymphoma (Figure 3A-3B). Of note, the vitreous biopsy obtained during the same surgery was found to be acellular.

**Figure 4:**
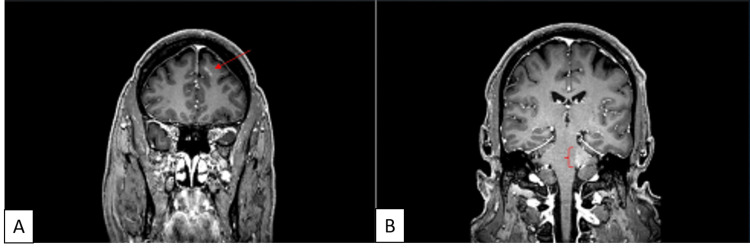
MRI of the Brain with Evidence of Intracranial Disease T1-weighted MRI of the brain with contrast showing a lenticular lesion within the left anterior frontal lobe (A, left) and a 2.3 x 1.6 cm lesion within the left cerebellar peduncle (B, right).

**Figure 5:**
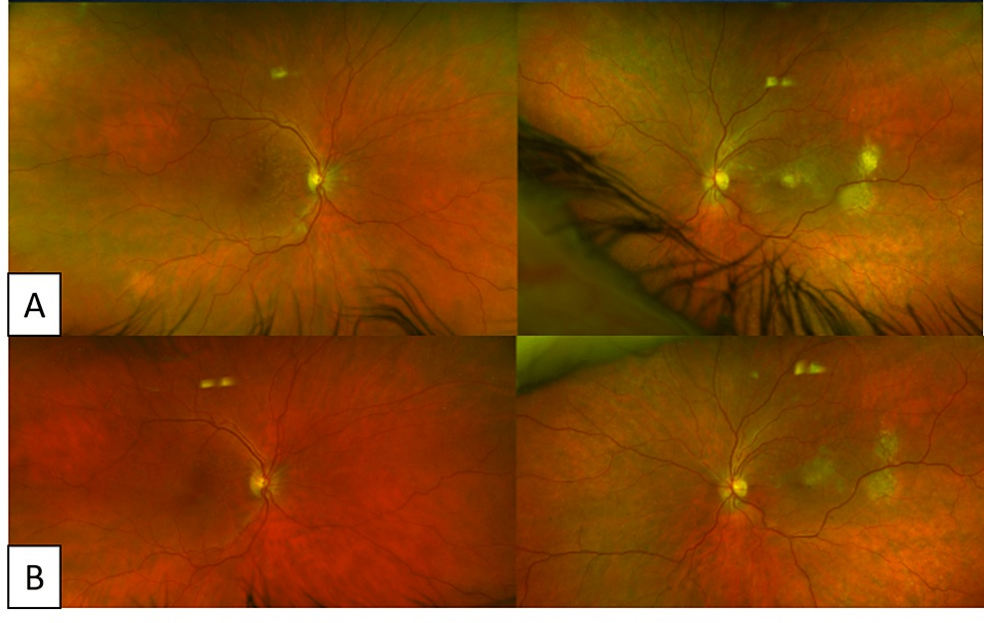
Serial Exams During Treatment Course Repeat imaging at one month (A) and three months (B) of systemic chemotherapy following treatment demonstrating excellent response.

The patient was initially given three methotrexate intraocular injections in the first two weeks following the diagnosis. An MRI of the brain revealed lesions in the left cerebellar peduncle and left frontal lobe (Figure 4A-4B). The patient was started on aggressive systemic chemotherapy with high dose methotrexate and cytarabine every three weeks (methotrexate 2000 milligrams IV over 24 hours on day 1, cytarabine 4 grams every 12 hours for four doses on day 2 and day 3, and leucovorin 400 milligrams IV every 6 hours for eight doses) to achieve CNS and intraocular penetration. Additionally, he was planned to undergo 17 treatments of whole-brain radiation. Ocular lesions were monitored while on systemic treatment and an excellent clinical response was achieved (Figure 5A-5B). By his one-month follow-up appointment, he had completed two cycles of chemotherapy and one treatment of whole-brain radiation. At that time, his uncorrected distance VA was 20/20 in the right eye and 20/25 with eccentric viewing in the left eye. He was examined every two months and found to have a stable appearance of his ocular lesions and stable visual acuity. His CNS lesions, however, continued to progress despite ongoing chemotherapy and whole-brain radiation. The patient died one year after his initial presentation to our clinic.

## Discussion

Primary vitreoretinal lymphoma (PVRL) is an ocular malignancy that is defined as a subset of primary central nervous system lymphoma (PCNSL) in which lymphoma cells are initially present only in the eyes without evidence of disease in the brain, spinal cord, leptomeninges, or cerebrospinal fluid [3-7]. PVRL follows a particularly aggressive clinical course and can be challenging to diagnose due to its ability to masquerade as noninfectious or infectious uveitis, or other neoplasms such as metastatic cancers [1]. The presentation of PVRL can be diverse and non-specific, with vitritis present in 66% of cases and prototypical cream-colored sub-retinal yellow infiltrates being reported in only 41% of cases [8]. These sub-retinal infiltrates can give rise to the characteristic “leopard skin” pigmentation of the fundus [9]. Many cases of PVRL are initially misdiagnosed as chronic posterior uveitis, and can respond to steroid therapy with immediate recurrence after tapering of the steroids. It is well-recognized, however, that corticosteroids are cytotoxic to PCNSL cells, and they appear to decrease the viability of tumor cells obtained in samples of vitreous and CSF [6]. This in turn leads to increased false-negative sampling. Even still, vitreous biopsies for PVRL are well-known to have high rates of false-negative sampling irrespective of concurrent steroid therapy. A study at the National Eye Institute of 12 patients demonstrated that 30% of cases of PVRL had had previous false-negative sampling [6]. Ophthalmologists are therefore encouraged to repeat vitreous cavity washout or pursue more aggressive biopsy techniques when suspicion of PVRL remains high in spite of a negative initial biopsy. Whitcup et al. emphasize that prompt and appropriate handling of specimens and review by an experienced cytopathologist are critical to the diagnosis of vitreoretinal lymphoma [6].

We elected to perform a 23-gauge pars plana vitrectomy with sub-retinal biopsy of one of the suspicious lesions to achieve the greatest diagnostic yield, as we felt that a vitreous biopsy alone would have low yield due to the vitreous being acellular. A limited flow cytometry analysis for the evaluation of B-cell antigens and light chains expression may prove extremely useful for diagnosis confirmation, as shown in our case. Of note, the vitreous biopsy obtained during the same surgery was found to be acellular.

PVRL occurs mostly in middle-aged and older adults, although patients as young as 15 years of age have been reported [7, 10]. When PVRL presents initially with ocular symptoms, the time to the development of central nervous system involvement can range from 7 months to 108 months with a mean of 29 months, although the disease can sometimes remain limited to the eye [11]. Progression from ocular disease to CNS disease has, however, been observed as quickly as one month [12]. Disease-free survival and overall survival at two years have been reported to be 20 and 40%, respectively [13]. The natural history of PVRL, therefore, is highly variable.

The progression of our patient’s ocular disease was far more rapid than the most conservative timelines described in the literature, based on serial fundus photography. He was found to have suspicious sub-retinal lesions in one eye with floaters and decreased vision three months following a normal funduscopic exam. Under the direction of a medical oncologist, he also had a normal MRI of the orbits and CSF studies within two weeks of presentation. Unfortunately, MRI of the brain was not obtained at that time; as such, we cannot fully exclude the possibility that brain lesions may have already been present. Five weeks after the initial presentation, he developed lesions in both eyes and brain lesions were identified on MRI of the brain. He was subsequently diagnosed with large B-cell lymphoma by sub-retinal biopsy.

Our patient’s time from presentation to ocular diagnosis of five weeks is also far shorter than the most conservative mean times to diagnosis described in the literature. A retrospective review of 83 HIV negative PVRL patients by Grimm et al. revealed a median time to diagnosis at 6 months and only 11 percent of the study patients were diagnosed with CSF studies, while Whitcup et al. reported that the mean time-to-diagnosis was 21.4 months [6,14]. It is not uncommon for patients to be followed up for years with a diagnosis of intermediate or posterior uveitis before a definitive diagnosis of PVRL is made through histopathologic evaluation of a vitreous specimen, a chorioretinal biopsy specimen, or an enucleation specimen, confirming the presence of lymphoma cells [6]. To our knowledge, the most rapid progression of primary vitreoretinal lymphoma described in detail was reported by Sangave et al., whose patient had a previous history of PVRL but developed rapid recurrence of her disease within two months of her most recent normal ocular examination, negative MRI, and negative CSF cytology [15].

## Conclusions

In conclusion, this case represents a very rapid intraocular progression of PVRL based on serial fundus photography - from unilateral, localized lesions to florid bilateral involvement and identification of CNS involvement in a five-week period despite the absence of vitreous involvement. Although the median time to diagnosis of PVRL is six months as reported by Grimm et al., it appears possible that a subset of PVRL patients may have particularly aggressive disease that can result in rapid progression and CNS spread in far less than six months. Therefore, the clinician should be mindful of the potential for rapid progression. In cases with high suspicion for PVRL, prompt vitreous and, if necessary, sub-retinal biopsy should be performed along with contrast-enhanced MRI of the brain.
